# Supplementary material for: All-silicon quantum light source by embedding an atomic emissive center in a nanophotonic cavity
Source: Nat Commun. 2023 Jun 7;14:3321. doi: 10.1038/s41467-023-38559-6 (PMC10247698; doi:10.1038/s41467-023-38559-6)
Supplement: Supplementary file 1 — Supplementary Information [file 41467_2023_38559_MOESM1_ESM.pdf]

Supplementary information for

All-silicon quantum light source by embedding an atomic emissive center in a nanophotonic cavity

W. Redjem[1*], Y. Zhiyenbayev[1*], W. Qarony[1*], V. Ivanov[2], C. Papapanos[1], W. Liu[2], K. Jhuria[2], Z. Y. Al Balushi[3,4], S. Dhuey[5], A. Schwartzberg[5], L. Z. Tan[5], T. Schenkel[2], and B. Kanté[1,4 †]

[1]*Department of Electrical Engineering and Computer Sciences, University of California Berkeley, Berkeley, California 94720, USA*
[2]*Accelerator Technology and Applied Physics Division, Lawrence Berkeley National Laboratory, Berkeley, California 94720, USA*
[3]*Department of Materials Science and Engineering, University of California Berkeley, Berkeley, California 94720, USA*
[4]*Materials Sciences Division, Lawrence Berkeley National Laboratory, Berkeley, California 94720, USA*
[5]*Molecular Foundry, Lawrence Berkeley National Laboratory, Berkeley, California 94720, USA*

[†] bkante@berkeley.edu

[*] These authors contributed equally to this work

Supplementary Note 1 – Optical properties of emissive centers in SOI bulk

1.1. Ensemble and single centers formation

To form silicon emissive centers, carbon ions are implanted in the 230 nm SOI wafer with a fluence of 4×10^{13} cm^{-2} and an energy of 36 keV. The parameters are chosen so that the implanted carbons are located at 100 nm in the silicon layer. After cleaning, the carbon-implanted wafer is annealed using rapid thermal annealing (RTA) with controllable annealing time and temperature.

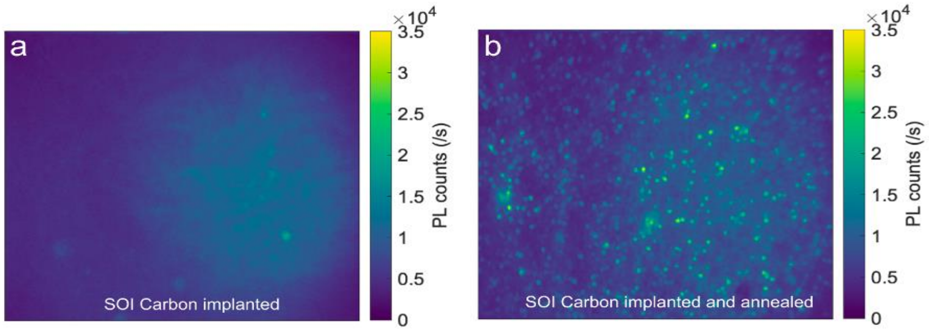

Supplementary Figure 1. Photoluminescence raster scanning of quantum centers. *Photoluminescence (PL) raster scans of* ***a,*** *carbon implanted samples and* ***b****, carbon implanted and annealed samples.*

Supplementary Figure 1a shows the photoluminescence raster scan of a sample that was only carbon implanted, and Supplementary Figure 1b corresponds to a sample that was carbon implanted and then annealed at 1000°C for 5 s. For the implanted sample, we observe a relatively uniform photoluminescence (PL) signal across the sample corresponding to the emission from an

ensemble of G-centers. The PL spectrum from the ensemble is shown in Supplementary Figure 2 and presents a zero-phonon line at 1278 nm along with a broad phonon sideband at higher energies. Implanted silicon wafers contain a concentration of carbon in the order of $5\text{x}10^{18}$ cm^{-3}, corresponding to 10^4 silicon atoms per carbon atom. From that, the density of defects for ensembles is estimated to be about >10-1000 G-centers/μm^2. Once the wafer is annealed, we observe bright individual spots that indicate the presence of single emissive centers (confirmed by autocorrelation measurements). For the same excitation power and same beam size, the single center is almost twice as bright as the ensemble (see Supplementary Figure 2 below). The lifetime of the ensemble of G-centers is 6 ns, while single centers have a lifetime of 35 ns. The density of single quantum emitters, estimated under the annealing condition of 5 s at 1000°C, is ρ_{QE} = 0.06 μm^{-2} corresponding to a single-center effective area of $1/\rho_{\text{QE}}$ = A_{QE} = 16.6 μm^2.

The process flow of the formation of emissive centers can be understood as follows. Implantation forms silicon self-interstitial [Si(*i*)] by moving substitutional carbon [C(*s*)] resulting in a high concentration of carbon interstitial [C(*i*)]. The C(*i*) has a low activation energy of about 0.8 eV and is very mobile at room temperature. The C(*i*) is then captured by a C(*s*) that is already present to form a dark carbon-carbon (CC) complex. The latter will transform into the bright B-form of the G-center composed of two C(*s*) and a Si(*i*) [1]. During annealing, C(i) are released from the centers, and the density of centers decreases. The thermal annealing is carefully optimized to increase the density of individually addressable centers (see Supplementary note 1.2).

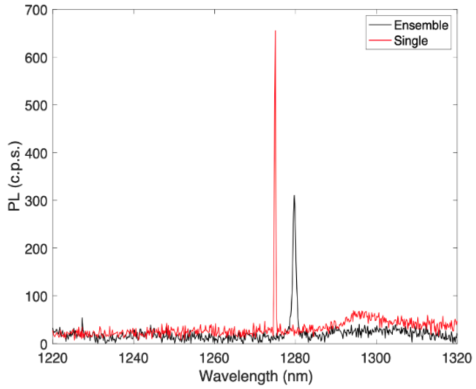

Supplementary Figure 2. Photoluminescence spectrum of an ensemble and a single center under the same excitation power and temperature*. Photoluminescence spectrum of an ensemble of G-centers (black) observed right after carbon implantation of an SOI wafer. The zero-phonon line is located at 1278 nm with a full width at half maximum of about 0.3 nm. Rapid thermal annealing at 1000°C for 5 seconds leads to the formation of single centers. The spectrum of a single G-center, shown in red, is blue-shifted with respect to the ensemble. At higher wavelengths, we observe phonon sidebands, corresponding to radiative transition involving electron-phonon coupling.*

1.2. The effect of annealing on the density and inhomogeneous broadening of single centers zero-phonon line

The density of single centers is investigated as a function of the annealing time in Supplementary Figure 3. We identify a time window below which only ensembles of centers are created (<3s) and beyond which all centers are thermally destroyed (>30 s). Within this time window, the density follows an exponential decay (solid red line) with a characteristic destruction time of τ=24.4±7.0 s. At 1000°C, the decrease in the density of centers is caused mainly by the release of C(*i*) from the G-center because of its lower activation energy (0.9eV) than C(s) and Si(i) (4eV). The G-centers transition from the B-form [consisting of two C(*s*)] to the A-form [consisting of one C(*s*) and one C(*i*)]. The C(*i*) is then released through a stochastic process. This is confirmed by the fact that the activation and migration energy of the C(*i*), which is 0.8 eV, is comparable to the thermal destruction energy of G-centers, which is around 0.9 eV.

Supplementary Figure 4 shows the distribution of the emission wavelength of approximately 150 single centers for a sample annealed at 1000°C for 5 s. For the sample annealed for 5 s, most centers emit at 1274 nm with a standard deviation of 4.7 nm. As we increase the annealing time (not shown here), the probability of emitting at a given wavelength decreases, and the Gaussian distribution becomes exponentially broader. The inhomogeneous broadening of the single centers is much larger than the one for the ensemble. The mean value of the ZPL emission of single emitters is always blue shifted as compared to the ZPL of the ensemble.

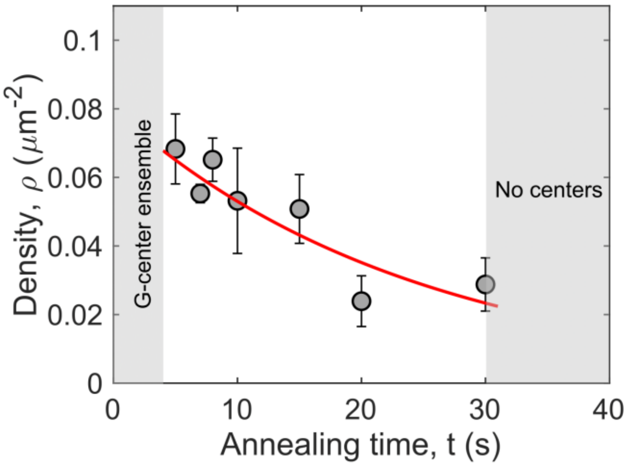

Supplementary Figure 3. Density of single G centers. *Density of single centers as a function of the annealing time at 1000°C. The density at 5 s annealing time is more than three times larger than the density for 20 s annealing. For an annealing time, shorter than 5 s, only an ensemble of centers is observed. For an annealing time longer than 30 s, all centers are destroyed.*

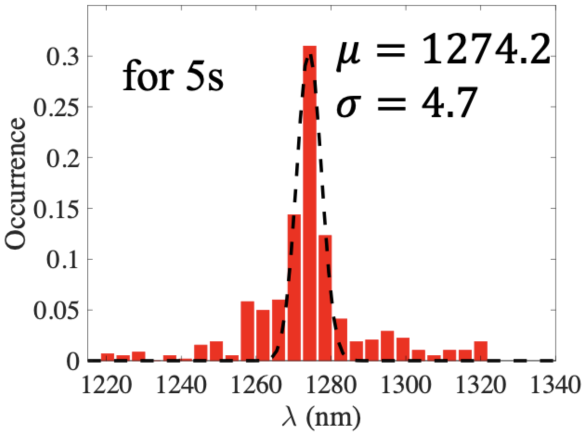

Supplementary Figure 4. *Distribution of the zero-phonon line emission of single emissive centers in silicon for an annealing time of 5 s at an annealing temperature of 1000°C.*

1.3. Polarization of the single quantum emitters

The fabrication of centers has been done using [100] SOI wafers. The in-plane dipole orientation is estimated by measuring the polarization diagram. We used a half-wave plate and a polarizing beam splitter before the detectors and recorded the PL counts as a function of the rotation of the half-wave plate. Results are presented in Supplementary Figure 5 and they show preferential orientation of the dipoles in two directions.

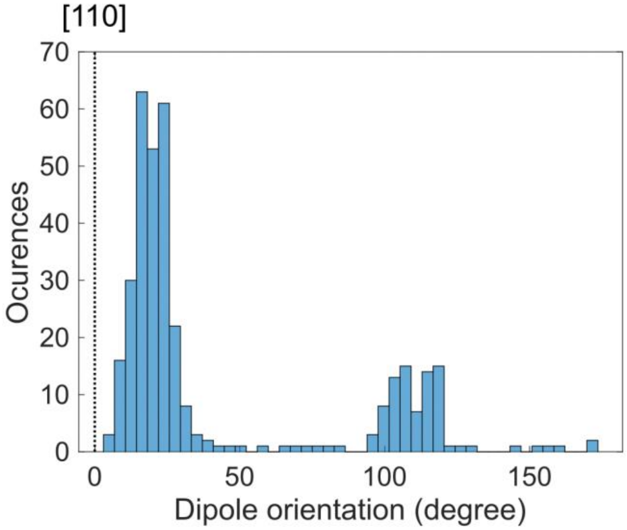

Supplementary Figure 5. *Dipole orientation distribution measured from single centers on SOI bulk carbon implanted and annealed at 1000°C for 5 s.*

Finally, a dipolar fit reveals the angle of the dipoles with respect to the [110] axis. The most prominent peak is slightly detuned from the [110] crystallographic axis of the silicon by 20 degrees. This information can be used to orient the photonic crystal cavity such that its

polarization has the same orientation as most of the defects present in the histogram. The standard deviation in the dipole orientation of the single emissive centers is about 15 degrees around the preferential direction.

1.4. Details of the first-principles calculations

We performed first-principles calculations of the silicon G-center (Type–B configuration) using the Vienna Ab initio Simulation Package (VASP). The color center defect was embedded within a 3 × 3 × 3 supercell of silicon and relaxed on a 2 × 2 × 2 k-point grid using the screened hybrid functional of Heyd, Scuseria, and Ernzerhof (HSE06) with an energy cutoff of 450 eV, converging to an energy tolerance of 10^{-10} eV and forces of 0.001 eV/Å. The excited state energy was computed using the k-point dependent constrained occupation approach, while the excited triplet state was obtained by constraining the spin occupation within VASP. VASPKIT toolkit was used to compute the transition dipole moment vector at the Γ point using the energy levels corresponding to the localized defect states [2]. In Supplementary Figure 6, we present the computed local structure of the G-center. The silicon and the two carbon atoms are located in the [100] mirror symmetry plane. We also computed the electronic wavefunction corresponding to the ground state and excited state of the defect. The orbitals of the excited state are strongly localized on the silicon self-interstitial.

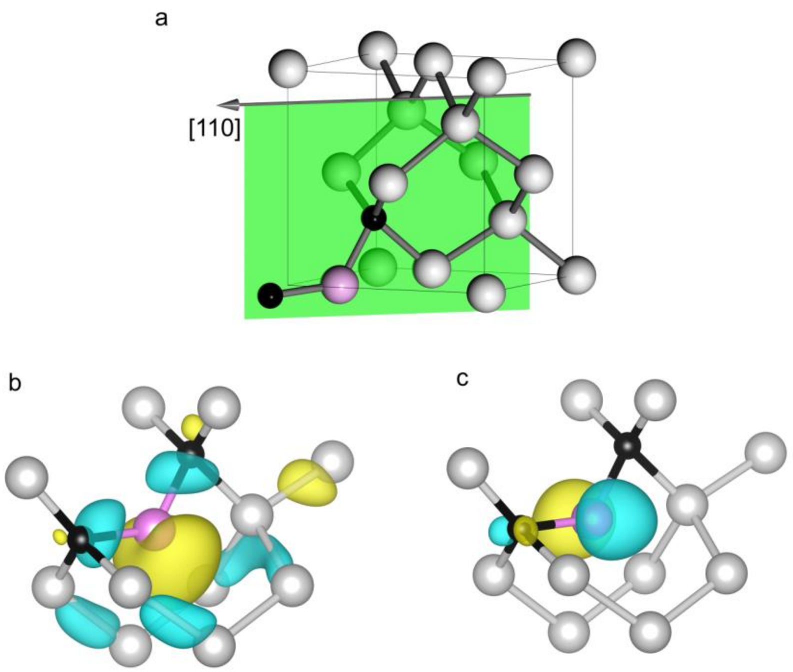

Supplementary Figure 6. *Local structure of the silicon G-center in the B-type configuration, composed of silicon (white) and carbon (black) atoms. The green plane in (a) marks the [110] mirror symmetry plane of the G-center. This plane contains all three atoms comprising the G-*

center - the two substitutional carbons (black), C(s), and the silicon self-interstitial (pink), Si(i). The real-space components of the wave functions corresponding to the lower (b) and upper (c) defect levels of the G-center are also presented, with yellow/blue denoting positive/negative amplitude.

To understand the origin of the broadening and shifting of the ZPL statistics, we computed the photoluminescence spectrum of the G-centers under strain [1]. We found that the volume expands by 1.22% for the isolated G-center compared to the volume in the presence of a nearby G-center. According to our calculations, expansion of the lattice corresponding to tensile strain would lead to a blue shift of the ZPL. Thus, the lattice is under high compression after implantation, and annealing releases some of the strain. However, since we used a very short annealing time, the lattice is not fully healed, and annealing would create more local disorder. This is because the annealing of silicon results in several microscopic processes which can affect the defect emission locally. For instance, we can have the release of Carbon interstitials from a G-center, the release of silicon interstitials and the formation of vacancy near a G-center, and/or incorporation of carbon interstitials into substitutional lattice positions near a G-center.

In the three processes discussed above, the silicon lattice locally contracts by a percent or less, leading to an overall "blue shift" of the emission. Moreover, the magnitude of the induced local strain is highly dependent on the distance between the G-center and the location of the microscopic process causing the strain. We notice that the strain can vary significantly depending on the location of the vacancy and may cause the observed large inhomogeneous broadening in the emission of single centers.

Supplementary Note 2 – Fabrication of single centers embedded in photonic crystal cavities

2.1. Carbon implantation and the depth distribution of carbon

Stopping and Range of Ions in Matter (SRIM) simulations are performed to determine the depth distribution of carbon in silicon. The simulations are conducted for a silicon-on-insulator (SOI) with a thickness of 230 nm of silicon and 400 nm of the oxide layer under a carbon implantation energy of 36 keV and a fluence of 4×10^{13} cm^{-2}. These parameters result in a maximum concentration of carbon around the middle of the silicon layer at a depth of about 100 nm as depicted in Supplementary Figure 7.

To confirm that the carbon is still located at a depth of about 100 nm in the silicon layer after the annealing step, we performed secondary ion mass spectrometry (SIMS) measurements that are also presented in Supplementary Figure 7. The SRIM and SIMS distributions match well, indicating that the diffusion of carbon during our annealing process is negligible.

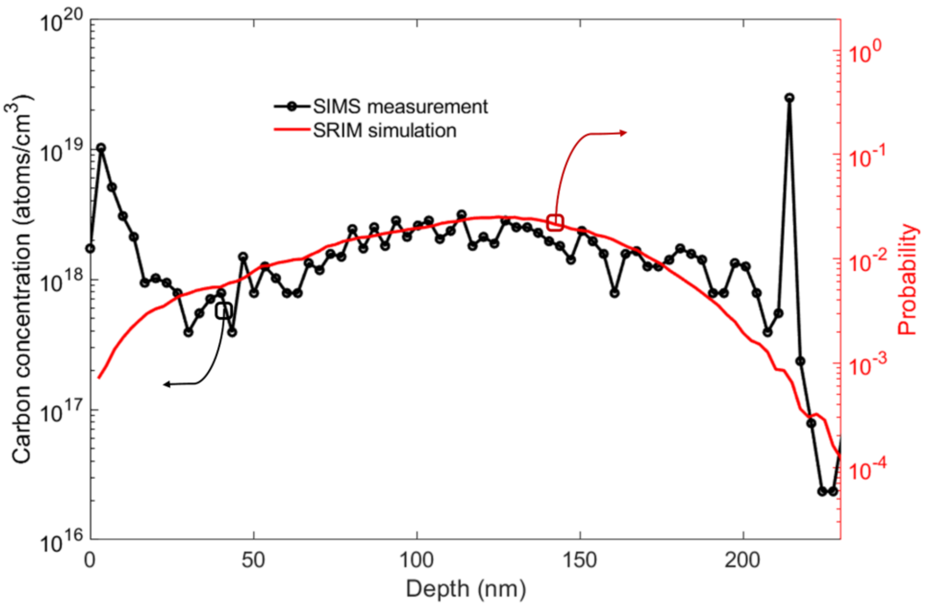

Supplementary Figure 7. Depth distribution of carbon in silicon. *The black curve (left axis) shows the concentration of carbon atoms after rapid thermal annealing at 1000°C measured by secondary ion mass spectroscopy (SRIM). The red curve (right axis) indicates the depth distribution of carbon obtained by Stopping and Range of Ions in Matter (SRIM) simulations. Even after annealing, the maximum carbon density is still located at about 100 nm, despite the diffusion process of carbon occurring during annealing.* ^{13}*C concentration spikes at 210 nm is an artefact at the interface Si/SiO2 due to ion yield change caused by the composition change in materials. The increased concentration close to the surface is due to diffusion of species during annealing at room temperature. The annealing at 1000°C that we perform does not affect the carbon profile concentration after implantation at 36 keV, because the concentration follows very well the SRIMS calculations (red line) that was done without considering the annealing process.*

2.2. Fabrication process flow

The implanted commercial SOI wafer is diced into a 1 cm^2 chip and cleaned using the RCA process. The chip is then UV exposed and baked to improve the adhesion for the resist. A positive tone electron beam (EB) photoresist is spun for e-beam lithography. Finally, the coated sample is soft baked in a controlled furnace.

In the next step, the samples are exposed to electron-beam lithography, followed by developing the EB resist. An inductively coupled plasma etching process defines the photonic crystal holes. A gas-based chemistry is used to etch the silicon with good vertical sidewalls and smoothness. Afterward, the EB resist is removed before a rapid thermal annealing process [3].

Usually, an ensemble of luminescent centers, such as W and G, are created during the dry etching of the silicon material [4]. We perform rapid thermal annealing to thermally destroy the ensembles and to form single-color centers with a high signal-to-background noise ratio in carbon-implanted samples. After the resist-stripping process, the sample is cleaned with a piranha solution for 10 min to confirm a residuals-free sample, including metals that are prone to damage during the rapid thermal annealing process.

Finally, a wet etching process is conducted by dipping the sample into a buffered oxide etchant solution (5:1) for 5 min, resulting in full suspension of the cavities. The samples are dried on a hotplate at 50°C after dipping them in an isopropanol (IPA) solution without breaking the membranes.

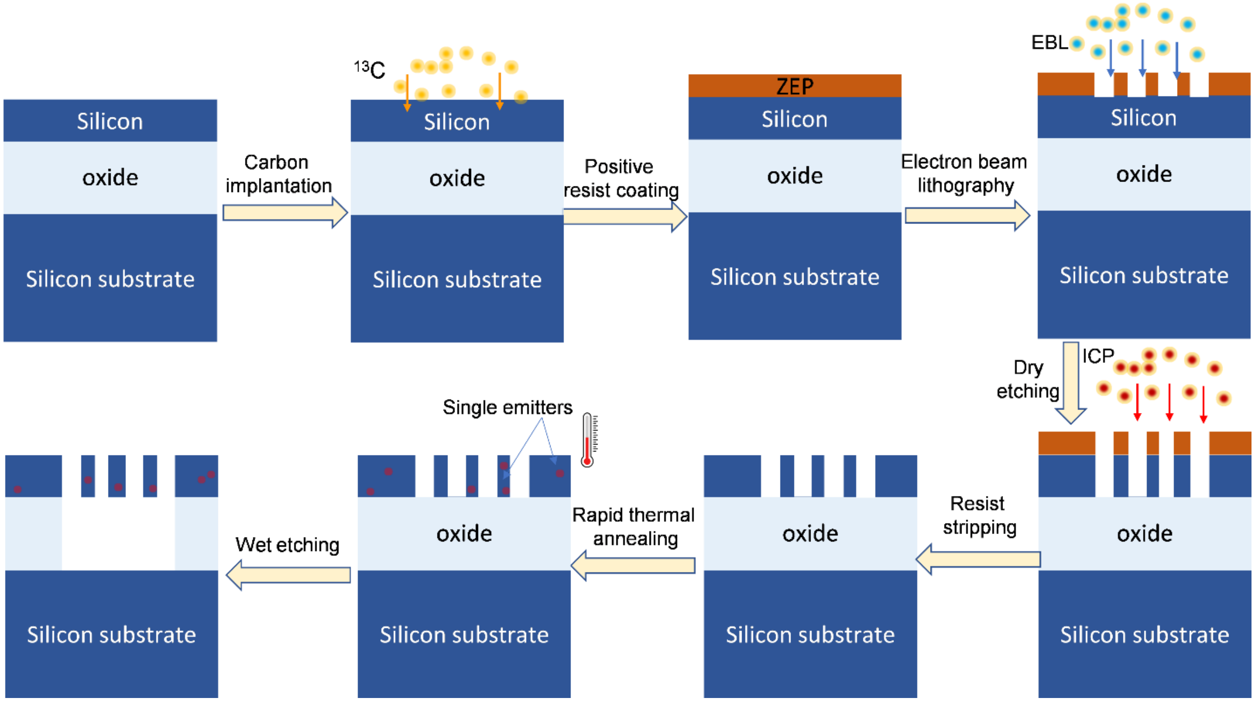

Supplementary Figure 8. Fabrication process flow of silicon-based cavities with silicon G centers. *A commercial silicon-on-insulator (SOI) wafer with a 230 nm silicon layer is carbon implanted at 36 keV with a fluence of* 4×10^{13} *cm*$^{-2}$*. G-centers are usually generated through the capture of carbon interstitials (*C_i*) introduced during the implantation and are thus not typically observed in natural silicon. After implantation, a high concentration of* C_i *is generated due to the formation of silicon self-interstitial* Si_i *that kicks out substitutional carbon* C_s*. The implanted wafer is diced into 1 cm*^{2} *chip, RCA-cleaned, exposed with UV light for 5 min, soft-baked at 180°C for 5 min, coated by ~150 nm of ZEP-502A positive tone electron beam resist, followed by baking at 180°C for 5 min (step 1 - step 3). The coated chip is exposed by electron-beam lithography and etched by an inductively coupled plasma dry etching process (step 4 - step 5). The ZEP-502A resist*

was stripped by 1-Methyl-2-pyrrolidone (NMP) for 15 min and annealed using a rapid thermal annealing processor at 1000°C for 5 s (step 6 - step 7). The chip is finally wet-etched to make a suspended membrane in the air. The fabrication process is optimized to create a good density of single centers coupled into the cavities.

2.3. Scanning electron microscopy images of the photonic crystal cavities

Supplementary Figure 9 shows the scanning electron micrograph of the final fabricated device, where the contrast between the exterior and the interior of the photonic crystal and elevated edges around the membrane designate the full suspension of the device. However, the SEM image of the devices before the wet etching process step does not pronounce such color contrast, indicating that the cavities are not suspended.

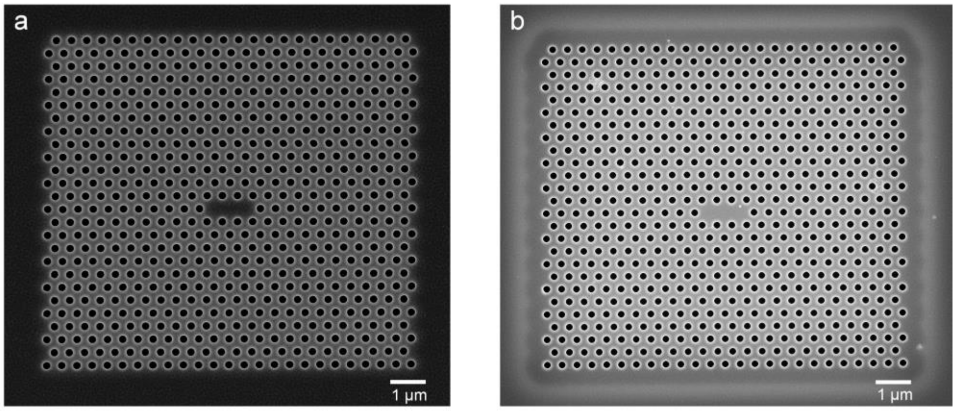

Supplementary Figure 9. SEM images showing non-suspended and suspended air cavities. *Scanning electron microscopic (SEM) images of (a)non-suspended cavities and (b) suspended in the air cavities. The color* contrast between the exterior and the interior of the photonic crystal and the elevated edges around the membrane in (b) designate the full suspension of the device, whereas (a) does not pronounce such color contrast as well as elevated edges around the membrane as the wet etching step was not performed.

2.4. Distribution of the ZPL of the quantum emitters and the resonance wavelength of the cavities

Supplementary Figure 10 shows the measured resonance wavelengths of 30 different cavities with the same design characterized by resonant scattering. The standard deviation in the resonance wavelength of the cavities is smaller than 2 nm. To optimize the probability of a successful device where a single center is coupled to the photonic crystal cavity, the cavity resonance distribution must match the peak probability of the quantum emitter zero phonon line. The cavity resonance is designed to be at 1270 nm, slightly blue-shifted from the peak

probability for the ZPLs. Gas tuning will be used later to redshift the cavity resonance. The peak probability of the ZPL is around 1275 nm.

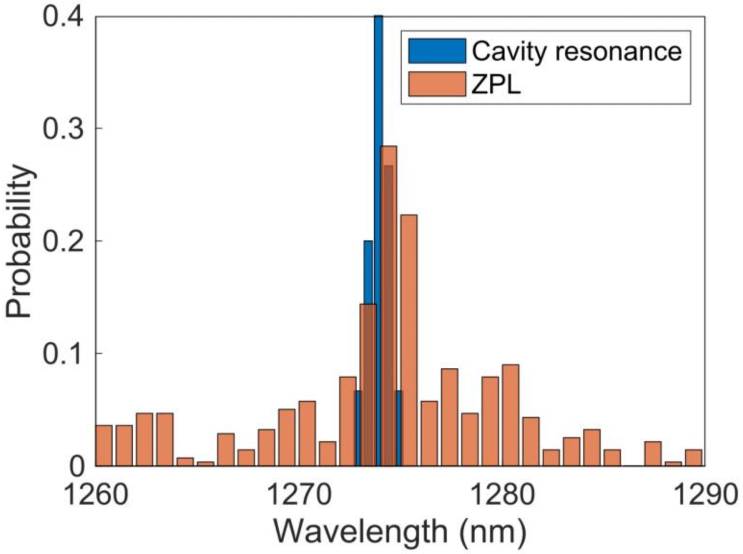

Supplementary Figure 10. *Distribution of the zero-phonon line emission of single centers in red, whereas the distribution of the cavity resonance is in blue.*

2.5. Statistics of cavity-enhanced single centers

Achieving high device yield is crucial for any scalable technique. To assess the effectiveness of our approach, we performed similar experiments using different devices. Supplementary Figure 11a represents photoluminescence spectra of cavities containing single centers on-resonance, along with their corresponding Q-factor values in Supplementary Figure 11b. Notably, the red spectrum at 1271 nm represents a single center located in the silicon bulk, and we observed a 2- to 13-fold enhancement in the zero-phonon line (ZPL) intensity with respect to the single defect located outside of the cavity (bulk). Supplementary Figure 11b shows the distribution of Q-factors, exhibiting an average value of 2525. Furthermore, Supplementary Figure 12 displays the raster scan images and the lifetime reduction of two other cavities (devices 2 and 3) in addition to one presented in the main text (device 1). As shown in the table below (Supplementary Table 1), we measured different Purcell factors. However, we have not yet identified any correlation between the Q-factor and the enhancement (i.e., the Purcell factor), as the latter is contingent on the relative position of the emitter within the cavity, which is probabilistic and uncontrolled in our approach.

	Lifetime ON (ns)	Lifetime OFF (ns)	Purcell factor	β factor, %
Device 1 (main text)	6.7	53.6	29.0	89
Device 2	6.2	23.5	26.1	79
Device 3	21.1	46.9	5.7	69

Supplementary Table 1: Summary of the Purcell factor/enhancement of three cavities containing a single center.

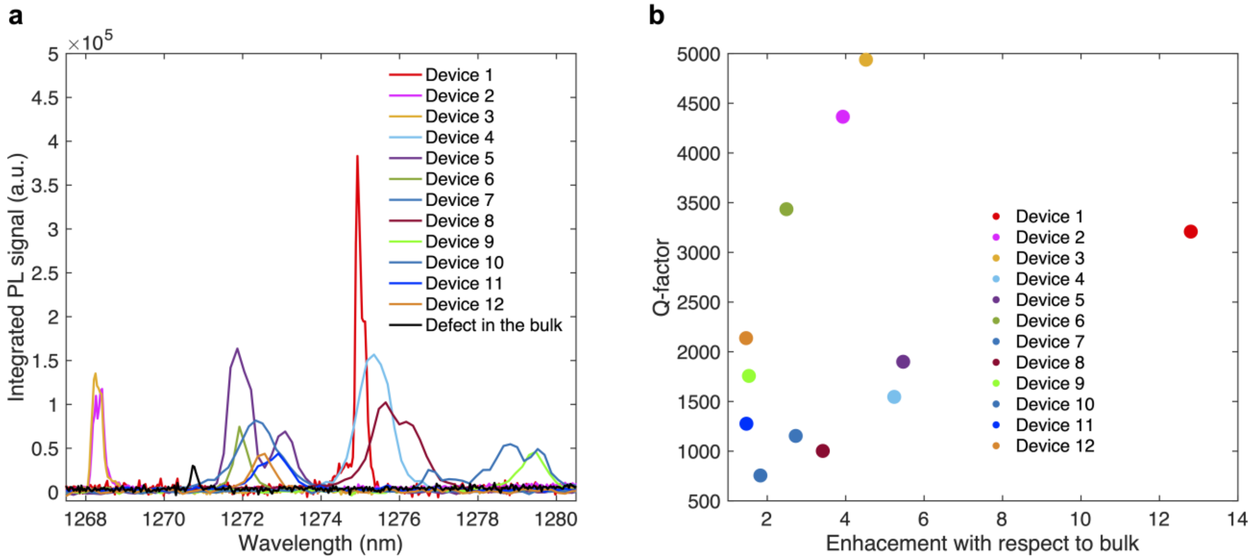

***Supplementary Figure 11**. **Statistics of the quantum emitters enhanced via the cavity interaction**. **a**, distribution of ZPLs enhanced by the cavity resonance. **b**, Distribution of the quality factors as a function of PL enhancement compared to the defect in the bulk.*

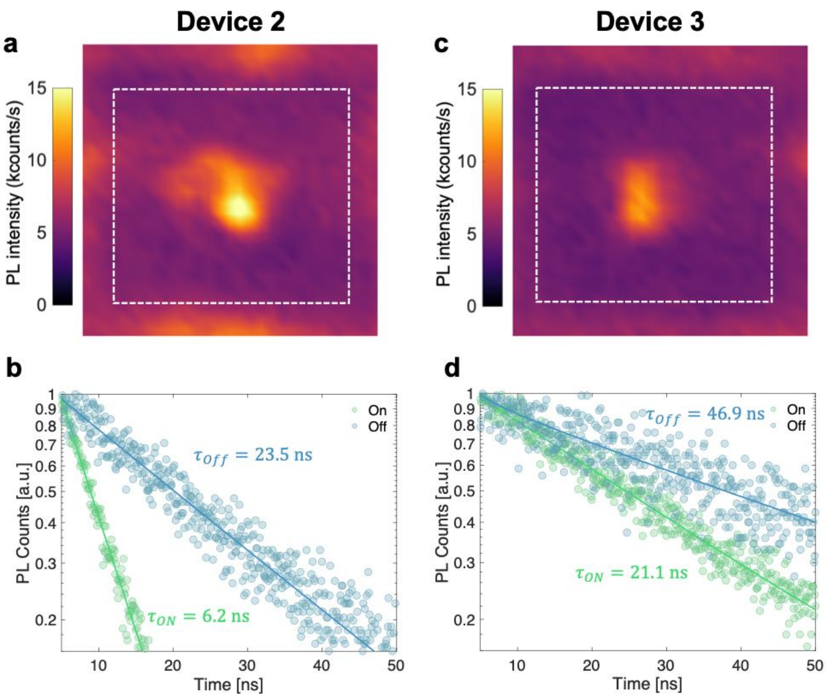

Supplementary Figure 12. Devices with enhanced and accelerated single photon emission. **a,c**, Raster scan images of the cavities (device 2 and 3) with a silicon defect located in the middle of the cavity region. **b,d**, Lifetime reduction of the emission in device 2 and 3 respectively.

Supplementary Note 3 – Experimental setup and measurements

3.1. Schematic of the experimental setup

Supplementary Figure 13 presents the experimental setup. The samples are placed in the cold platform (7K) inside a cryostat. The sample is optically excited using either continuous or pulsed green laser excitation at 532 nm. The pulsed laser has a variable repetition rate (100 kHz - 80 MHz) and a pulse width of 50 ps. The optical path of the pump laser beam is represented by a green line in Supplementary Figure 13. A dichroic mirror (DM) transmitting at 1000 nm is used to separate the pump beam and photoluminescence (PL) signal, shown in orange. A steering mirror, along with the two lenses (L), forms a telecentric system and allows for the precise addressing of a single defect by the laser beam.

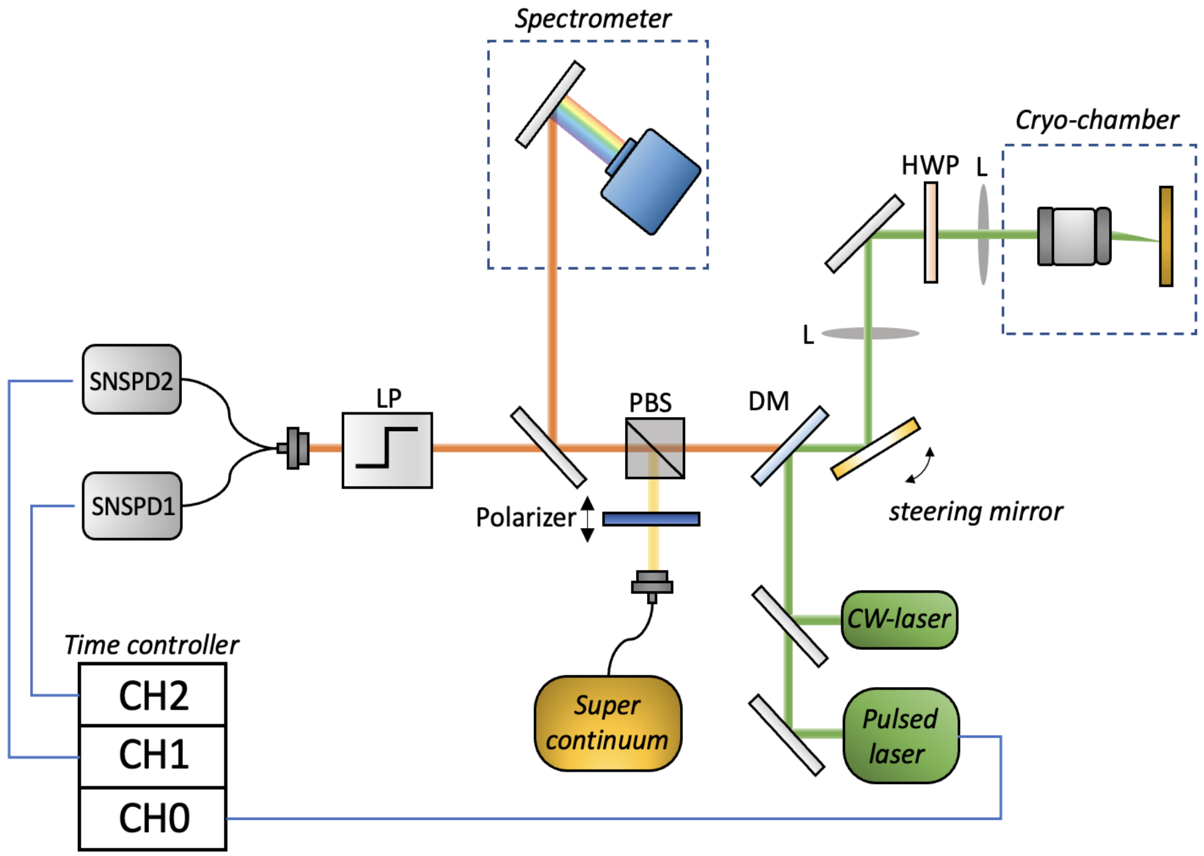

***Supplementary Figure 13. Schematic of the experimental setup**. The experiment is conducted at a low temperature of 4 K, which is achieved using a closed-cycle cryogenic chamber. The optical path of the 532 nm laser excitation, which can be either continuous or pulsed, is shown by a green line. The PL signal is separated from the pump beam using a dichroic mirror (DM) at 1000 nm. The long-pass filter at 1250 nm is used to increase the signal-to-noise ratio. The combination of a steering mirror and two lenses (L) separated by a distance of two focal lengths is used for raster scanning the sample. Two superconducting nanowire single-photon detectors (SNPDs) are used for photon counting and time-correlation measurements. The signal spectrum is analyzed using an InGaAs camera out-couple to a grating spectrometer. For reflectivity measurements, we used a supercontinuum white light source (400 - 2000 nm), along with a linear polarizer and a polarizing beam splitter (PBS). A half-wave plate (HWP) is used to rotate the polarization of the incident light to match the polarization of the nanophotonic cavity.*

The signal is coupled to a fiber beam splitter connected to two superconducting nanowire single-photon detectors (SNSPD). In addition, the long-pass filter (LP) at 1250 nm is used to suppress the remaining laser excitation and luminescence from the silicon. The coincidences counts are recorded with a time controller.

For the autocorrelation measurements, we monitor the coincidences between CH1 and CH2. The $g^{(2)}(\tau)$ data was normalized using to the following expression:

$$g^2{}_{norm}(\tau) = g^2{}_{exp}(\tau)/\Delta \cdot R_1 \cdot R_2 \cdot T, \qquad (1)$$

where, Δ is the bin width, *R1*, and *R2* are the count rate of the two detectors, respectively, and *T* is the total acquisition time.

We excited the center with relatively high power (about $3.3P_{saturation}$) to get the maximum counts. At high power, there is also a repumping mechanism that attenuates the bunching due to the metastable state [5].

For the time-resolved photoluminescence, the coincidences are recorded between CH0, which is connected to the trigger of the pulsed laser, and CH1.

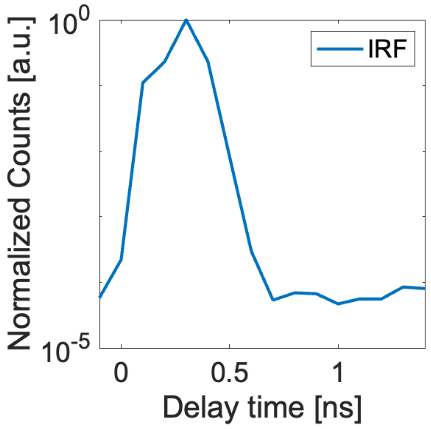

Supplementary Figure 14. *The response function from the time correlated instruments containing the time controller and the Superconducting nanowire single photon detectors. The time response is about 500 ps. The function was measured by sending an ultrafast laser pulse (100 fs at 1300 nm) to the detectors.*

The collected signal can also be directed toward the spectrometer comprising three types of gratings 150, 600, and 1200 g/mm. The spectrum is measured with a thermoelectrically cooled InGaAs camera.

For resonance scattering measurements, we used a supercontinuum white light source that provides a continuous broadband light source from 400 to 2000 nm. The white laser is polarized vertically using a linear polarizer and reflected off to the sample through a polarizing beam splitter. A half-wave plate (HWP) positioned before the objective lens is rotated so that the incident polarization makes an angle of 45 degrees with respect to the cavity axis. The polarization of the scattered light that has been coupled to the cavity has no longer the same polarization as the incident light. The resonant scattered signal then passes through the PBS and is analyzed with the spectrometer.

3.2. Photon extraction efficiency

We estimated the total photon collection efficiency ($\eta_{extraction} \times \eta_{objective}$) by performing finite-difference time-domain simulations. The extraction efficiency (the first term) was obtained by placing a single dipole within the cavity and calculating the percentage of photons emitted into the far field. The objective-related efficiency (the second term) was determined by the numerical aperture of the objective lens. The resulting total collection efficiency as a function of the numerical aperture is shown in the Supplementary Figure 15 below. In our experiment, with NA=0.85, the total extraction efficiency was approximated as 30%, meaning that 30% of the photons emitted by the coupled emitter (with a near-unity coupling efficiency) were collected by the experimental setup.

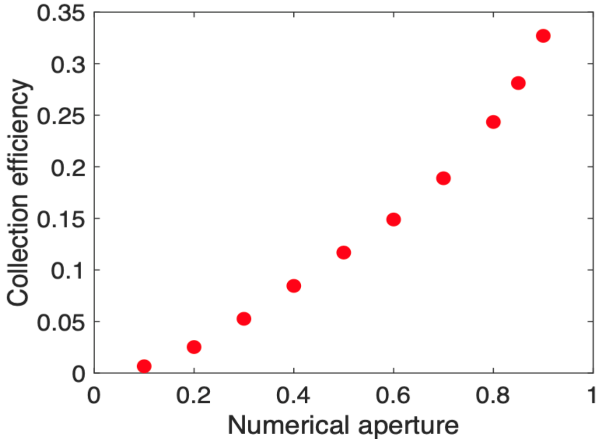

Supplementary Figure 15: *Collection efficiency as a function of the numerical aperture of the objective lens.*

3.3 Detuning-dependent Purcell factor

The experimental Purcell factor (circle) calculated from the expression is presented in the figure (Supplementary Figure 17) below:

$$F_p{}^{exp} = (\tau_{bulk}/\tau(\delta) \ - \tau_{bulk}/\tau_{OFF} \ )/\eta, \qquad (2)$$

Where $\tau(\delta)$ is the life as a function of the detuning, and the other terms are explained in the text. The black solid line corresponds to a Lorentzian of the form,

$$F_p^{theory} = 3Q/(0.66 \times 4\pi^2) \cdot \xi^2 \cdot \frac{\Delta\omega^2{}_{cav}}{4\delta^2 + \Delta\omega^2{}_{cav}} \qquad (3)$$

Where Q is the measured quality factor of the cavity, δ is the detuning of the cavity resonance, and $\Delta\omega_{cav}$, is the linewidth of the resonance of the resonator. ξ is the normalized dipole

orientation factor (Equation 6). In the figure below, the corresponding black line was plotted with a value of $\xi = 0.28$. The center is not positioned at the maximum of the optical field. We clearly see that Equation 3 (black line) matches well with the experiments and proves that the lifetime reduction is due to Purcell enhancement.

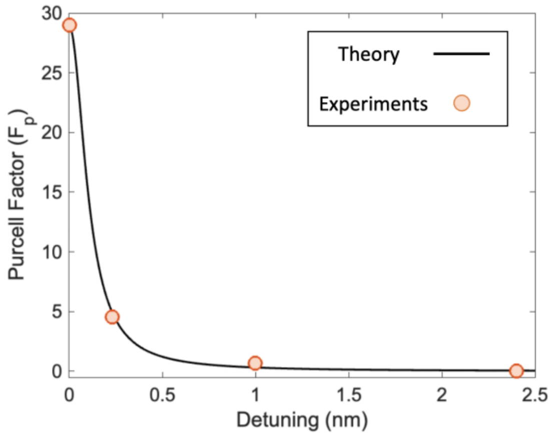

Supplementary Figure 16*: Purcell factor as a function of detuning. The black line is the theory (Equation 3) and the orange sphere is the experimental data extracted from the equation in the main text.*

3.4. Gas tuning of the cavity resonance

Fine-tuning of the cavity resonance is achieved by changing the effective index of the cavity mode. To change the index and control the resonance wavelength of the optical mode we condensate an inert gas on top of the sample [6]. For each cycle, a small amount of gas is injected inside the chamber. The gas condensates and freezes once in contact with the cold sample at a temperature of 4K. The solidified layer of Ar changes the effective index and red shifts the cavity's resonance. The amount of gas injected is controlled using a mass flow controller. Supplementary Figure 17a shows a typical gas-tuning experiment. In Supplementary Figure 17a, we clearly see that the resonance is red-shifting as we increase the amount of injected gas. The exact values of the gas tuning parameters can vary because the direction and rate of gas deposition depend on the position of the injection gas tube that can slightly change from one experiment to the other. The resonance wavelength of the cavity is controlled in Supplementary Figure 17b as a function of the cycle number. We achieved up to 8 nm shifts in the resonance wavelength.

When the surface of the photonic crystal layer is completely covered by sufficiently thick condensed argon gas, the resonance does not shift anymore. We also measured the quality factor of the nanophotonic cavity as we increased the number of cycles (Supplementary Figure 17b).

Initially, the quality factor is around 3500 and it drops continuously to 2000 when the number of cycles increases.

The process of gas injection can also be reverted by either hitting the sample with a high-power laser or by heating the sample platform above 85K. In both cases, the Ar gas evaporates progressively, and the cavity resonance shifts back to its original wavelength.

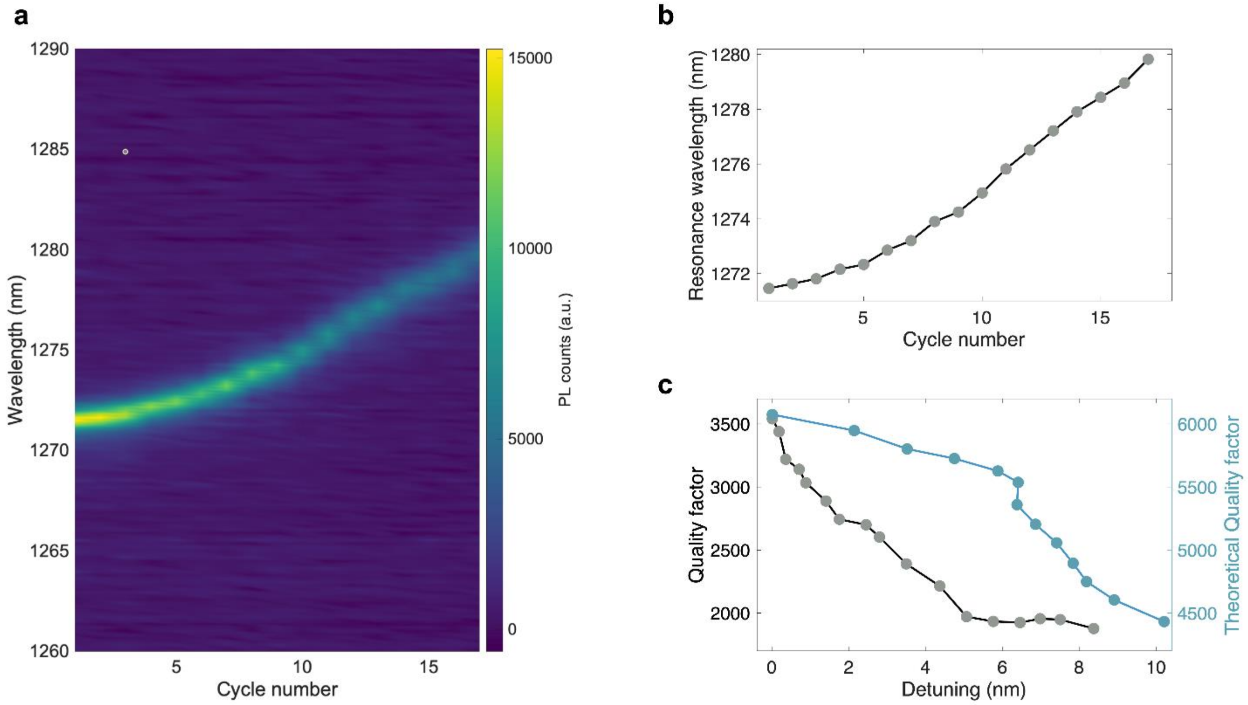

Supplementary Figure 17. *Gas injection-assisted cavity resonance tuning.* ***a****, Photoluminescence (PL) spectrum as a function of the gas injection cycle.* ***b****, Resonance wavelength tuning for increasing cycle number.* ***c****, Evolution of the quality factor of the nanophotonic cavity as the resonance wavelength is tuned. The computed quality factor is shown in blue (right axis).*

Supplementary Note 4 – Photonic cavity design

4.1. Purcell factor in optical microcavities

The radiative decay rate of an emitter in a cavity is governed by [7],

$$\gamma_{cav} = \frac{g^2\kappa}{\kappa^2+\delta^2}. \qquad (4)$$

The decay rate of the emitter in the cavity γ_{cav} is enhanced when $\delta = \omega_{QE} - \omega_{cav}$, the detuning between the quantum emitter (QE) emission frequency ω_{QE} and the cavity resonance (ω_{cav})

approaches zero. The decay rate can be enhanced by the Purcell effect as $\gamma_{cav} = F_p\gamma_0$, where γ_0 is the theoretical free space decay rate of the emitter, and the Purcell factor (F_p) is given by [7],

$$F_p = \frac{3Q(\lambda/n)^3}{4\pi^2 V_o}\xi^2 \frac{(\Delta\omega_{cav})^2}{4\delta^2+(\Delta\omega_{cav})^2}. \qquad (5)$$

Here λ is the free-space wavelength of the light, n is the refractive index of silicon, $\Delta\omega_{cav}$ is the resonance linewidth of the cavity and,

$$\xi = \frac{|p\cdot E(r_{QE})|}{|p||E_{max}|}, \qquad (6)$$

is the normalized dipole orientation factor with p being the dipole moment of the QE, $E(r_{QE})$ is the electric field at the position r_{QE} of the QE, and E_{max} is the maximum local electric field inside the resonator. The key to getting to a large Purcell factor is to optimize the normalized dipole orientation ξ and the dipole position.

4.2. The band gap of the infinite photonic crystal

The photonic crystal consists of a periodic arrangement of holes in a triangular lattice. The unit cell of the photonic crystal and its dispersion is presented in Supplementary Figure 18. The PhC has a complete gap centered around 1300 nm with a width of 284.3 nm. The optical density of states in the gap is zero, and the emission from centers within the photonic crystal is inhibited [8].

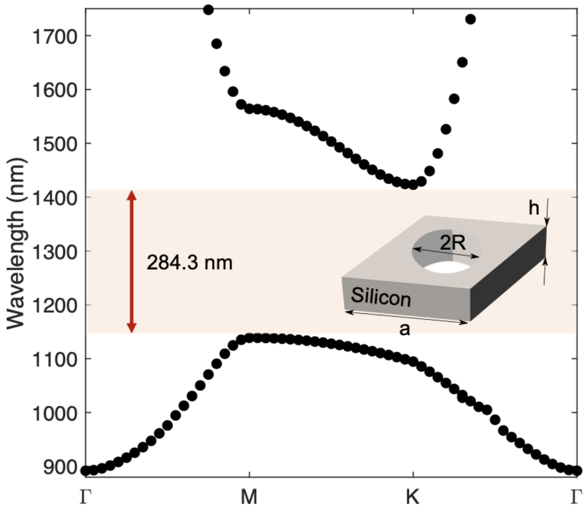

Supplementary Figure 18. *Band diagram for the transverse-electric (TE) optical modes of a triangular lattice PhC computed using a three-dimensional finite-element solver. The filled area denotes the photonic band gap centered at 1281.0 nm, and it spans 284.3 nm. The inset shows a*

sketch of the unit cell. The period of the lattice is a = 346.5 nm, the radius of the air hole is R = 96 nm, and the silicon membrane thickness is h = 230 nm.

4.3. Design of the photonic crystal defect cavity

To form the nanophotonic cavity, three holes are removed along the Γ-K direction of the PhC. The missing holes create localized states at frequencies within the photonic band gap. The band gap prevents the propagation of electromagnetic waves inside the lattice and confines the energy in the region of missing holes. As a result, the modes occupying these localized states exhibit high-quality factors and small mode volumes. This cavity, presented in Supplementary Figure 19, is also known as the L-3 defect cavity [8].

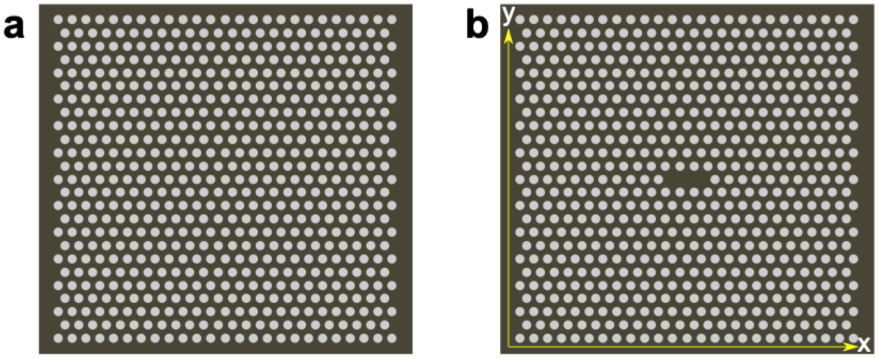

Supplementary Figure 19. Schematic of the photonic crystal structure. a, Triangular lattice of air holes. b, Photonic crystal defect cavity formed by three missing sites along the Γ-K direction in the center of the structure (L-3 cavity). The localized states emerge within the photonic band gap. The ratio between the radius and period of the lattice determines the wavelength of the resonance.

4.4. 3D Finite size simulations

The performance of the device is computed using finite-difference time-domain simulation in three dimensions. To compute the modal volume that can be calculated using,

$$V_{mode} = \frac{\int \varepsilon |E|^2 dV}{max(\varepsilon |E|^2)}, \qquad (7)$$

we positioned a narrow-band dipole source in the middle of the cavity. The computed electric field of the optical mode is shown in Supplementary Figure 20 and gives a mode volume $V_{mode} \approx 0.66(\lambda/n)^3$ for our cavity. The computed quality factor of our cavity is Q=6072. Finally, the theoretical Purcell factor of the emitter in the cavity (F_p) was estimated to be 700 using,

$$F_{p,theor} = \frac{3}{4\pi^2}\frac{Q}{V_{mode}}\left(\frac{\lambda}{n}\right)^3. \qquad (8)$$

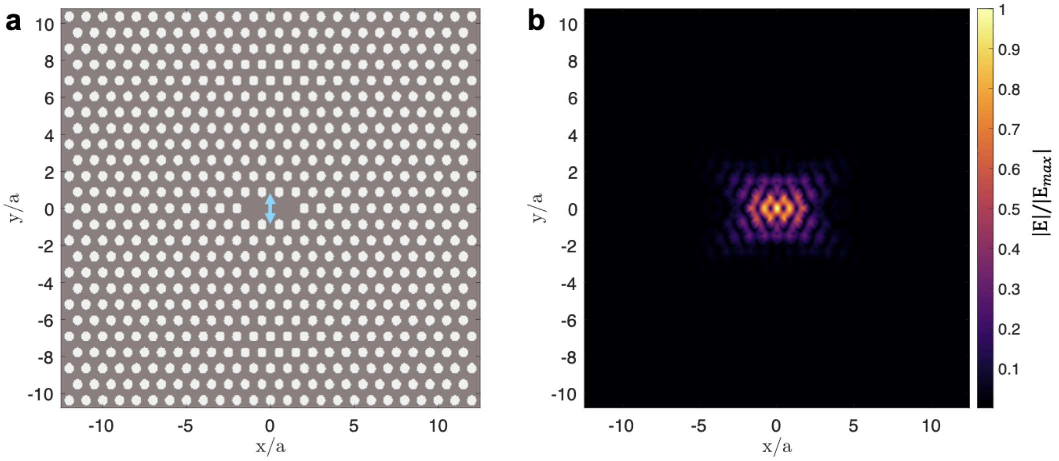

Supplementary Figure 20. *Time-domain computation of the electromagnetic mode in the defect cavity. a, Sketch of the photonic crystal cavity containing 25 unit cells along the Γ-K direction. The blue arrow in the center of the cavity indicates the location and orientation of the dipole source. b, Magnitude of the electric field of the fundamental L3-cavity mode excited by an electric dipole using a three-dimensional finite-difference time-domain numerical simulation. The electric dipole is placed at the antinode of the electric field. The electric field is oriented parallel to the plane of the membrane (TE-mode).*

4.5. Dipole-dipole alignment in the Purcell effect

The Purcell factor dramatically depends on the modal volume, quality factor, and dipole moments alignment (see Equations 5 and 6). The Purcell enhancement as a function of the rotation of the in-plane dipole with respect to the cavity axis is shown in Supplementary Figure 21. As expected, the evolution of the Purcell factor depends on the square of the sinus of the angle between the cavity and dipole emitter moment. A significant Purcell factor is achieved when the dipole moment of the emitter is perpendicular to the cavity axis, and it vanishes when the dipole moment is along the cavity axis.

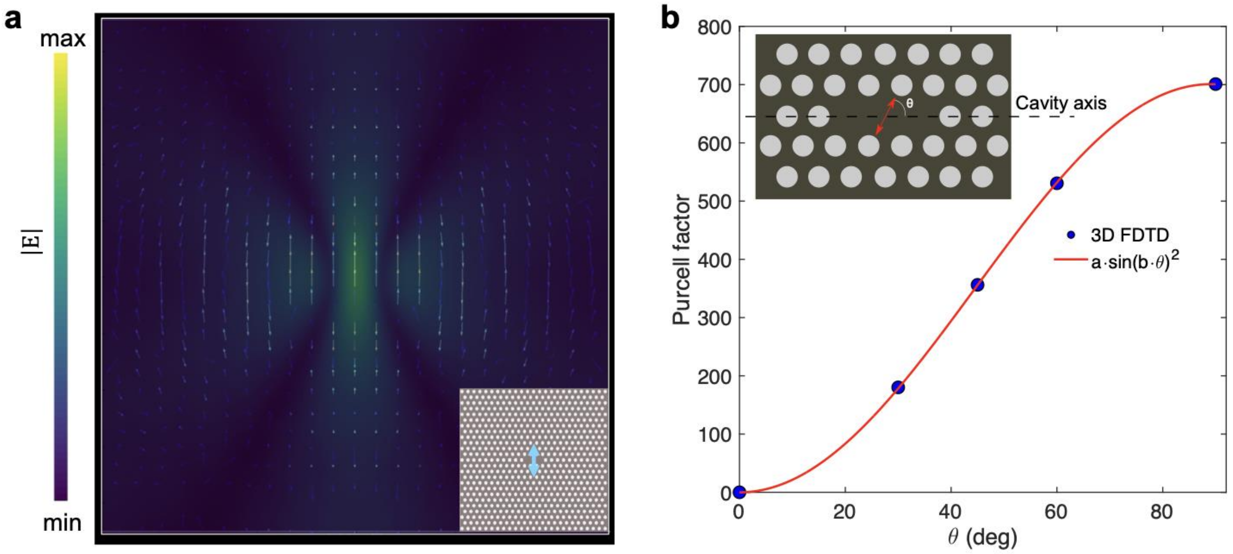

Supplementary Figure 21. *Polarization of the localized electromagnetic mode.* ***a****, Computed electric-field distribution (magnitude of the electric field) detected by a field monitor above the cavity. The arrows indicating the direction of the electric field reveal that the cavity is linearly polarized. The inset shows the dipole source orientation inside the cavity.* ***b****, Purcell enhancement for different orientations of the electric dipole. Numerical simulation is fitted by a squared sinusoidal function.*

References:

[1] Zhiyenbayev, Y. *et al.* Scalable manufacturing of quantum light emitters in silicon under rapid thermal annealing. *Opt. Express, Vol. 31, Issue 5, pp. 8352-8362* **31**, 8352–8362 (2023).

[2] Ivanov, V. *et al.* Effect of localization on photoluminescence and zero-field splitting of silicon color centers. *Phys. Rev. B* **106**, 134107 (2022).

[3] Campbell, S. A. . The science and engineering of microelectronic fabrication. vol. 164 (Oxford University Press, 2001).

[4] Weber, J. et al. Photoluminescence detection of impurities introduced in silicon by dry etching processes Appl. Phys. A 41, 175–178 (1986).

[5] Redjem, W. *et al.* Single artificial atoms in silicon emitting at telecom wavelengths. *Nat. Electron. 2020 312* **3**, 738–743 (2020).

[6] Mosor, S. *et al.* Scanning a photonic crystal slab nanocavity by condensation of xenon. *Appl. Phys. Lett.* **87**, 141105 (2005).

[7] Loudon, R. Quantum mechanics of the atom-radiation interaction. *Quantum Theory Light* 46--81 (2000).

[8] Sakoda, K. Optical Properties of Photonic Crystals. **80**, (2005).
